# Acquired lymphedema: Molecular contributors and future directions for developing intervention strategies

**DOI:** 10.3389/fphar.2022.873650

**Published:** 2022-10-25

**Authors:** Ika Nurlaila, Kangsan Roh, Chang-Hwan Yeom, Hee Kang, Sukchan Lee

**Affiliations:** ^1^ Department of Integrative Biotechnology, Sungkyunkwan University, Suwon, South Korea; ^2^ Department of Vaccine and Drugs, The National Research and Innovation Agency, Jakarta, Indonesia; ^3^ Cardiovascular Research Center, Massachusetts General Hospital, Harvard Medical School, Boston, MA, United States; ^4^ Division of Cardiology and Corrigan Minehan Heart Center, Massachusetts General Hospital, Harvard Medical School, Boston, MA, United States; ^5^ Yeom Chang-Hwan Hospital, Seoul, South Korea; ^6^ Humanitas College, Kyung Hee University, Yongin, South Korea

**Keywords:** lymphedema, therapy, phytochemicals, adipogenesis, fibrosis, animal models

## Abstract

Lymphedema is a debilitating chronic disease that mostly develops as an adverse reaction to cancer treatment modalities such as chemotherapy, surgery, and radiotherapy. Lymphedema also appears to be a deteriorating consequence of roundworm infections, as best represented by filariasis. According to its origin, lymphedema is classified as primary lymphedema and acquired lymphedema. The latter is an acquired condition that, hitherto, received a considerably low attention owing to the less number of fatal cases been reported. Notably, despite the low mortality rate in lymphedema, it has been widely reported to reduce the disease-free survival and thus the quality of life of affected patients. Hence, in this review, we focused on acquired lymphedema and orchestration of molecular interplays associated with either stimulation or inhibition of lymphedema development that were, in vast majority, clearly depicted in animal models with their specific and distinct technical approaches. We also discussed some recent progress made in phytochemical-based anti-lymphedema intervention strategies and the specific mechanisms underlying their anti-lymphedema properties. This review is crucial to understand not only the comprehensive aspects of the disease but also the future directions of the intervention strategies that can address the quality of life of affected patients rather than alleviating apparent symptoms only.

## 1 Introduction

Lymphedema has been characterized as a morbid chronic disease, that is, typically developed following cancer treatment, mainly chemotherapy (Kim et al., 2015), radiotherapy (Daley et al., 2010; Kunkler et al., 2015), and surgery (Garza et al., 2017), and has affected 130–250 million people worldwide (Park et al., 2013). Lymphedema represents an adverse yet inevitable pathophysiological response to treatment against specific cancers, particularly breast cancer (Fu, 2014; McDuff et al., 2019), and gynecologic cancer (Lim et al., 2014; Rowlands et al., 2014; Biglia et al., 2017; Saner et al., 2019). It is a clinical manifestation of impaired lymphatic drainage (Biglia et al., 2017), which leads to the accumulation of interstitial fluid with high protein content in the affected subcutaneous tissues (Bok et al., 2018) and further induces stiffness and thickness of the tissue microstructures (Lee et al., 2020). Despite having infrequent mortality, it lowers quality of life because of the co-occurring disabilities of the affected or adjacent parts of the body, particularly the limbs. Although surgical and post-surgical therapies have enormously advanced worldwide, lymphedema is poorly overcome and limitedly managed (Saito et al., 2013). To date, no drug specifically targeting lymphedema has been licensed (Bruns et al., 2003; Gardenier et al., 2017), suggesting that the development of an efficacious anti-lymphedema is has a long way. However, according to the findings of a few *in vivo* and *in vitro* studies, altering the status of lymphedema from incurable to curable chronic disease is not impossible. Therefore, in the present review, we highlight primary molecular events or characteristics of acquired lymphedema, particularly in its association with anticancer treatments, and update to the most recent progress regarding some phytochemical-based substances that exhibit anti-lymphedema properties. Overall, the present review depicts acquired lymphedema as a long-due challenge and shows some promising opportunities for less-explored substances to contribute profoundly to the global search for specific and efficacious drugs to reverse or regulate the contra-productive effects of the disease.

## 2 Secondary (acquired) lymphedema

According to its origin or initial cause, lymphedema is classified as 1) primary lymphedema and 2) secondary (acquired) lymphedema, the latter of which is the focus of this article. However, we have introduced primary lymphedema also.

Primary lymphedema is a rare condition that affects approximately 1.2 patients in 100,000 patients aged below 20 years (Maclellan and Greene, 2014). This is associated with several gene mutations such as *VEGFR-3* (Milroy disease), *SOX18* (hypotrichosis-telangiectasia-lymphedema), *CCBE* (Hennekam syndrome), and *FOXC2* (lymphedema distichiasis) (Maclellan and Greene, 2014; Kambhampati et al., 2016). Primary lymphedema may occur in different age groups. Congenital lymphedema occurs at an age of up to 2 years, whereas lymphedema precox and lymphedema tarda occur at an age below and above 35 years, respectively (Kambhampati et al., 2016). Although lymphedema has been initially recognized prior to more than a century, understanding of its causal aspects is considerably less progressive (Karkkainen et al., 2000).

Unlike primary lymphedema, which is a clinical manifestation of insufficient lymphatic transport, acquired lymphedema is a consequence of traumatic perturbation of the lymphatic system, mainly due to malignancy, inflammation, and infections (Rutkowski et al., 2006; Padera et al., 2016; Borman, 2018; Hong et al., 2019; Bertelli et al., 2020). Acquired lymphedema constitutes more than 90% cases of lymphedema worldwide (Greene and Goss, 2018/04).

In order to provide adequate consideration for intervention strategies, the International Society of Lymphology (ISL) has classified lymphedema based on superficial physical appearances into three stages (International Society of Lymphology, 2020) as described in Table 1. Each stage carries a particular level of severity according to the volume differences measured in the affected limb. It is considered minimal when the increase is less than 20%, moderate when the volume increases by 20%–40%, and severe when the limb volume rises by more than 40% (Tretbar et al., 2008). Lymphedema diagnosed at an early stage is conventionally treated with combined physical therapy to alleviate edema. However, this treatment is less longer effective for an advanced stage due to the presence of adipose tissue deposition (Hoffner et al., 2018) and accumulation of collagen and fibrotic tissue (Cho et al., 2017) which is irreversible and can only be removed by surgery (Hoffner et al., 2018). Anticancer treatment-induced lymphedema is barely reversible (Paskett et al., 2012), whereas obesity-induced counterpart lymphedema might have less detrimental effects when the body weight is maintained under the right proportion (Nitti et al., 2016). This leaves us with a major query on what builds up the gap between these differences with respect to the emergence of pathological features and the recovery between the two most common inducers of acquired lymphedema. Hence, we put our best efforts to tailor several prior-reported pathways associated with the development of acquired lymphedema. We also discuss the progress made so far with regard to animal models of lymphedema, one of which was developed in our laboratory. This is crucial to support the future studies focused on formulating the best and practical intervention strategies to alleviate the impacts of lymphedema in the clinical realm.

**TABLE 1:** Description of lymphedema staging.

Stage	Clinical description
Stage 0	• Swelling does not surface yet changes in the tissues’ compositions and compromised lymphatic transport are evident subtly
	• It can be transitory prior to edema
Stage I	• Relatively higher levels of protein-rich interstitial fluids than that for venous edema are observed
	• Proliferating cells might also be seen
Stage II	• Changes are more prominent in solid structures
	• Subcutaneous fat and fibrosis have started developing
Stage III	• Further accumulation of fat and fibrosis
	• Warty overgrowth
	• Alterations in skin characteristics and thickness

## 3 Clinical hallmarks of lymphedema

### 3.1 Perturbed lymphatic vasculature

The human lymphatic system is made up of lymphatic vessels, lymphatic organs, interstitial fluid, and migrating cells (Rovenská and Rovenský, 2011; Gavins et al., 2020). However, although the building blocks are diverse, the system works as a whole, and is an important part of the immune system (Rovenská and Rovenský, 2011).

The lymphatic vasculature is found in most organs and comprises lymphatic capillaries, collecting vessels with valves, and lymph nodes. The lymphatic capillaries are blind-ended and highly permeable, lined by a single layer of loosely connected endothelial cells; they merge into the collecting vessels and then into the thoracic duct and right lymphatic duct, and eventually empty lymph into the venous system (Oliver et al., 2020). Lymphatic development involves modification of a subpopulation of embryonic venous endothelial cells in the cardinal veins expressing prospero-related homeobox 1 (PROX1), a master transcription factor, into lymphatic endothelial cell (LEC) progenitors, which subsequently bud off from cardinal veins to form lymph sacs (Oliver et al., 2020). The activation of PROX1 induces chicken ovalbumin upstream promoter-transcription factor 2 (COUP-TF II) (Lee et al., 2008/09) and SRY box transcription factor 18 (SOX18) to initiate differentiation of LECs (Srinivasan et al., 2010). After forming the sacs, LECs sprout to produce peripheral lymphatic network, which is involved in regulation of the entire growth and remodeling of the lymphatic system (Escobedo and Oliver, 2017; Kim and Song, 2017; Oliver et al., 2020).

The lymphatic system is responsible for fluid transportation and maintenance of the overall fluid balance in the body. In the interstitial space, the fluid is at sub-atmospheric pressure. However, to reach the venous system it requires a pressure of approximately 20 cm H_2_O. This pressure difference is overcome by active pumping of collecting lymphatic vessels. In addition, passive vessels lead to further pumping (Moore and Bertram, 2018). However, a few causes or risk factors may impair this flow system, leading to the increased volume of interstitial fluid due to either increased inflow or decreased outflow or both; thus, the fluid balance is compromised (Ridner, 2013). This emerges as swelling (edema) and is the most common clinical symptom of lymphedema (Maclellan and Greene, 2014; Azhar et al., 2020) against which potential drugs/medicines are formulated.

### 3.2 Impaired lymphangiogenesis mediated by the VEGF-C/VEGFR-3 signaling pathway

Lymphangiogenesis is a process in which new lymphatic vessels are generated either from pre-existing lymphatic vessels or initial formation during embryogenesis (Varner and Schwab, 2011). This is a dynamic process during embryogenesis, but is almost absent postnatal under normal physiological conditions and only occurs in pathological conditions such as tissue remodeling, inflammation, and tumor growth (Christiansen and Detmar, 2011). It was highly associated with unfavorable clinical outcomes in tumors, particularly with respect to the recurrence-free survival rate and tumor size (Sha et al., 2019).

Under physiological conditions in adults, the lymphatic network system is remodeled into mature collecting vessels owing to the strong expression of VEGF-receptor-3 (VEGFR-3) in luminal valves and less strong in lymphangion. VEGFR-3 is a cognate ligand of vascular endothelial growth factor-C (VEGF-C) (Norrmén et al., 2011). Hence, the VEGF-C)/VEGFR-3 signaling pathway is apical to lymphangiogenesis (Veikkola et al., 2001). VEGF-C is a VEGF/platelet-derived growth factor (PDGF) family homolog with cysteine-rich C-terminal. Once it is proteolytically processed, it binds to VEGFR-3, previously termed as Fms-like tyrosine kinase-4 (FLT4) and induces autophosphorylation (Joukov et al., 1996). VEGFR-3 expression is detected in LECs, whereas VEGF-C is chemostatic and expressed by immune cell infiltrate, which is primarily a macrophage subset (Gousopoulos et al., 2017). It was reported that in mouse tail lymphedema model, VEGF-C was overexpressed by CD68^+^ cells, a macrophage marker, leading to the worsening of lymphedema’s most distinctive symptom, edema, and this was positively correlated with vascular leakage that initiated the fluid influx into the tissue. Blocking VEGF-C abolished edema development and vascular leakage (Gousopoulos et al., 2017).

VEGFR-3 alone was shown to be sufficient to induce lymphangiogenesis, as suggested by Veikkola et al., who developed transgenic mice overexpressing a VEGFR-3-specific mutant of VEGF-C (VEGF-C156S) or VEGF-D in epidermal keratinocytes using the keratin 14 promoter. In this study, lymphatic vessels in the skin were observed to be induced by both transgenes without affecting the architecture of the adjacent blood vessels (Veikkola et al., 2001).

During the state of lymphedema, lymphangiogenesis is perturbed, which results in the accumulation of protein-rich interstitial fluid, and thereby swelling, in certain sites of the body. Subsequently, the fluid induces inflammatory responses, which further initiate fibrosis, adipocyte deposition, and impaired immune response and wound healing (Norrmén et al., 2011). Therefore, in the treatment of cancer, where lymphangiogenesis facilitates the spread of cancerous cells into lymph nodes, should be abrogated; therefore, anti-inflammatory drugs such as docetaxel are generally used as a therapeutic strategy. However, the use of docetaxel, being toxic, as an adjuvant chemotherapy has been reported to be associated with a high risk of lymphedema in some breast cancer cohorts (Harris et al., 2018). This is intriguing yet conflicting because lymphangiogenesis is unfavorable in cancers but favorable in lymphedema; moreover, we agreed that lymphedema itself is an adverse consequence of anticancer treatment as described earlier in this review. This strongly indicates a potential molecular crosstalk between lymphangiogenesis and tumorigenesis (Gomes et al., 2013; Lee et al., 2015), which is of major interest, particularly with regard to developing drugs against cancer without triggering lymphedema or drugs that negate the emerging clinical hallmarks of lymphedema (Anton and Rockson, 2019). As described earlier that lymphangiogenesis is initiated through the VEGF-C/VEGFR-3 signaling pathway, VEGF-C and VEGFR-3 are two main targets proposed in both synthetic-based and phytochemical-based drug platforms, which are discussed in this article (6. Phytochemical-based substances with anti-lymphedema properties).

A selective VEGFR-3 tyrosine kinase inhibitor, SAR131675, was shown to inhibit lymphangiogenesis in both *in vitro* and *in vivo* studies through regulation of the pro-inflammatory chemokine-expressing M1 macrophages, which further leads to decreased fibrosis (Hwang et al., 2019). Although the VEGF-C/VEGFR-3 axis is crucial for lymphangiogenesis to enable the trapped lymphatic fluid to re-flow properly, this might trigger a contra-productive clinical manifestation since lymphangiogenesis is supportive for cancer metastasis. This is certainly of concern for researchers and clinicians worldwide because the vast majority of patients with acquired lymphedema are receiving treatment for various stages of cancer. This urges further studies focusing not only on the crosstalk of lymphangiogenesis in simultaneous cancer and lymphedema, but also on drugs targeting other than lymphatic-related development. Anti-fibrosis and anti-adipogenesis are the two most proposed strategies, which are elucidated in the following sections.

### 3.3 Excessive fibrosis

In addition to lymphangiogenesis, fibrosis is a major hallmark of acquired lymphedema. Fibrosis and lymphangiogenesis are in opposite direction. While lymphangiogenesis is favorable for alleviating lymphedema, fibrosis is one of critical players in lymphedema. Both differ in terms of dependency on the VEGF-C/VEGFR-3 gradient. Fibrosis is a manifestation of excessive accumulation of extracellular matrix, mainly collagen, which subsequently harden the sites of wounds or injuries, inducing chronic inflammatory responses (Wynn, 2008). In contrast to lymphangiogenesis, that is, greatly suppressed in lymphedema and occurs due to the involvement of both VEGF-C and VEGFR-3, fibrosis was modulated in a VEGF-C-independent manner as demonstrated by Avraham et al. (2009) and earlier by Rutkowski et al. (2006), where only weak expression of VEGF-C was detected in the swollen tail of murine models (Avraham et al., 2009). Fibrosis is widely known as a key inhibitor of the tissue fluid accumulation which causes tissue expansion. This is a barrier to lymphangiogenesis (Avraham et al., 2009). It causes accumulation of collagen (Ghanta et al., 2015) and α-smooth muscle actin (α-SMA) around the podoplanin-expressing collecting lymphatic vessels (García Nores et al., 2018) takes a longer time than that of the interstitial fluid which results in a delayed lymphedema (Ghanta et al., 2015).

Fibrosis development is strongly associated with the M2-phenotype of macrophages (Ghanta et al., 2015) and Th2-skewed inflammatory response of CD4^+^ T cells (García Nores et al., 2018). M2-macrophages promote fibrosis by releasing transforming growth factor (TGF)-β1 as a profibrotic cytokine (Ghanta et al., 2015). In addition, they produce cytokines such as IL-13, IL-10, IL-4, tumor necrosis factor-α (TNF-α), and IL-1 (Van Linthout et al., 2014) which synergistically activate fibroblasts, recruit myofibroblasts, and exacerbate inflammatory cell infiltration to the injured site (Van Linthout et al., 2014; Ghanta et al., 2015). TNF-α, in particular, is shown to positively loop feedback the production of TGF-β1 *via* a kinase-specific pathway. However, the nuclear factor-κB (NF-κB) pathway is profoundly involved in the production of pro-inflammatory cytokines (Van Linthout et al., 2014). The significance of M2-macrophages in fibrosis is evident as their depletion ameliorates fibrosis. Notably, the depletion is selective and not general; during progressive injury, macrophage depletion leads to reduced fibrosis (Duffield et al., 2005), which is a favorable clinical feature in any of the proposed anti-lymphedema strategies. However, in the recovery state, this depletion is unable to overcome the recruitment of cellular and matrix components of the fibrotic response (Duffield et al., 2005), thus increasing fibrosis and accumulation of CD4^+^ T cells. In contrast, VEGF-C expression is weakened, suggesting macrophages as a source of VEGF-C along with LECs (Ghanta et al., 2015).

The CD4^+^ T cell subset plays a crucial role in fibrosis similar to that in M2-macrophages. This perspective was triggered following a critical finding by Nierdermeier et al. (2009) that fibrocyte differentiation was determined by the presence of CD4^+^ T cells. Fibrocytes are collagen-type I producing hematopoietic cells. Activation of the CD4^+^ T cell subset initiates the release of some soluble cytokines such as TNF, IL-4, IFN-γ, and IL-2, which prevent massive growth of fibrocytes. Interestingly, in the presence of calcineurin inhibitors and absence of mTOR inhibitors, when CD4^+^ T cells were in their activation state, marked collagen I deposition was detected, indicating the steady growth of fibrocytes. These results suggest that the activation of CD4^+^ T cells determines the fate of fibrocytes. This finding was supported by Tapmier et al. (2010), who demonstrated that depletion of CD4^+^ T cells using monoclonal antibodies was observed to significantly decrease the interstitial expansion and collagen deposition in a ureteric-obstructed RAG knock-out murine model setting. As such, CD4^+^ T cell blocking is considered a potential strategy for anti-fibrosis intervention. More recently, collagen III was also indicated to play a role in fibrotic skin of grade 3 lymphedema with the gene transcription level was detected much higher up to 39 fold than that seen for collagen I (Karayi et al., 2020). This bulks up potential targets of anti-lymphedema intervention although much more in-depth studies are necessitated to discern underlying mechanisms of both activation and regulation of collagen III from gene to protein levels and potential functional exchange between collagen I and collagen III.

In the global efforts to seek a cure for acquired lymphedema, the overall orchestration of fibrosis has recently received a lot of attention. Unlike targeting lymphangiogenesis, which not only allows re-building of the previously impaired lymphatic system but also re-promote cancer cell metastasis (Schlereth et al., 2014), targeting fibrosis is considered more appealing because it is an unfavorable prognostic factor for both cancer and lymphedema; hence, fibrosis suppression is expected to improve the outcomes for patients with cancer (Chandler et al., 2019) as well as for those suffering from lymphedema post cancer treatment (Gardenier et al., 2017; Mehrara et al., 2021).

### 3.4 Adipogenesis

One aspect that causes acquired lymphedema to be less likely, if not impossible, to cure is its progression cycle. The regions initially affected by lymphedema expand outwards to the skin through neighboring fatty tissues, resulting in concomitant changes in the lymphatic system and adipose tissue that must be focused to address lymphedema (Tashiro et al., 2017).

Adipose tissue is a lobulated septal network niched to diverse cell types, including adipocytes, fibroblasts, vascular endothelial cells (VECs), vascular smooth muscle cells, and immune cells mainly macrophages and lymphocytes (Tashiro et al., 2017). As represented by specialized organelles inside adipocytes, adipose tissue is responsible for the storage of triglycerides and cholesterol esters in the form of lipid droplets. Therefore, changes in the amount of lipids stored in adipocytes affect the fat cell size (DM et al., 2007). Adipocytes, the predominant cells residing in adipose tissue, are generated through a process termed adipogenesis, in which differentiation and maturation of adipocytes from mesenchymal stem cells (MSCs) are facilitated (Lane and Lennarz, 2013). This is a biphasic process and necessitates cell-cell communication *via* surface molecules, extracellular matrix, and direct contact. When preadipocytes completely develop into adipocytes, the cells use extracellular matrix to make envelops; thus, direct cell-cell contact is no longer possible (Schling and Löffler, 2002).

Transcriptional regulators modulate Adipogenesis. Zfp423 is a multi-zinc finger transcription factor that accounts for cell commitment to the adipogenic lineage (Gupta et al., 2012) and has exceptionally dense CpG sites in its promoter (Yang et al., 2013). This factor regulates peroxisome proliferator-activated receptor-γ (PPARγ), which is required for the formation of white and brown adipose tissue in the body, and CCAAT-enhancer-binding protein (C/EBPs) to facilitate differentiation of preadipocytes into adipocytes (Gupta et al., 2012). Zfp423 activation is facilitated *via* the bone morphogenetic protein (BMP)/*Cae* *norhabditis elegans* SMA and mothers against decapentaplegic (SMAD) signaling pathway (Bond et al., 2018) and the overall functions are mainly controlled by Wnt1-inducible signaling pathway protein 2 (WISP2), which forms a complex with the Zfp423 molecules in the cytoplasm. BMP/SMAD signaling enables dissociation of the WISP2-Zfp423 complex and Zfp423 enters the nucleus. Subsequently, transcriptional activation of some downstream genes is triggered (Yang et al., 2013; Bond et al., 2018; de sá et al., 2017).

In acquired lymphedema, adipogenesis is dramatically upregulated, causing the affected tissue to be suffused with adipocytes, further upregulating fat differentiation markers (Azhar et al., 2020) such as PPARγ, C/EBP-α/β, and FABP4 (Lowe et al., 2011; Ji et al., 2014; Roh et al., 2017a; Roh et al., 2020; Koc et al., 2021). Therefore, adipogenesis, along with other superficial clinical hallmarks, is a promising target for the treatment of lymphedema. These ideas have attracted increased attention after the achievement of successful treatment along with prolonged survival rate and improved prognosis by blocking or modifying adipogenesis activation-associated markers in some cases of cancer and obesity, particularly in women (Yang et al., 2013; Wang et al., 2017; Wu et al., 2019; Oshi et al., 2021).

## 4 Risk factors of lymphedema

### 4.1 Anticancer treatments

#### 4.1.1 Chemotherapy

Chemotherapy is, thus far, the mainstay of cancer treatment; however, in some cases, it fails to elevate the quality of life of the patient receiving chemotherapy. Chemotherapy is associated with the fluid retention in extremities. Although direct mechanisms linking chemotherapy and emergence of lymphedema is yet to be delineated but the association of both is evident, particularly that of taxane-based (Qin et al., 2011; Swaroop et al., 2015) which was reported to increase 2-years accumulative incidence of lymphedema to 22.76% which was much higher than the incidence found in non taxane based treatment (Swaroop et al., 2015). Hidding et al. (2018) demonstrated that after completion of adjuvant treatment with docetaxel, doxorubicin, and cyclophosphamide, breast cancer patients developed lymphedema and experienced significant decline in physical function because of the increased swelling, heaviness, and volume changes in the upper arm. Similar concerns were also reported for patients receiving paclitaxel (Swaroop et al., 2015). Later, Kim et al. reported an increased propensity of node-positive breast cancer patients treated with adjuvant chemotherapy across types (Kim et al., 2015).

Despite these widely reported long-term adverse effects, chemotherapy is still regarded as the most common regimen for both solid and blood cancers because it is undeniably capable of inducing cell death *via* apoptosis, programmed cell death, or necrosis characterized by accidental cell death. While apoptotic cancer cells are specifically and markedly detected by tagging small portions of the cells with a few sets of molecular markers, the necrotic counterpart cells lack these markers (Ricci and Zong, 2006). Nonetheless, necrotic cell death activates tissue inflammation in the adjacent tissue in response to the release of intracellular materials termed damage-associated molecular patterns (DAMPs). Subsequently, pro-inflammatory cytokines, chemokines, and adhesion molecules are produced (Yang et al., 2015); hence, the balance of Th1/Th2 immune response is perturbed and skewed into Th2 that triggers a phenotypic switch of macrophages to M2 macrophages (Dijkgraaf et al., 2013). Following this shift, either VEGF-C or VEGFR-3 is downregulated and lymphatic function is impaired (Yeh et al., 2017; Tacconi et al., 2019).

Macrophages are immune cell subsets that are markedly upregulated at the site of lymphedema (Duan et al., 2016). Macrophages polarize into either pro-inflammatory M1 macrophages or anti-inflammatory or reparative M2 macrophages, based on the circulating signals they receive from their microenvironment (Mishima et al., 2017). Among these two phenotypes, M2 macrophages are responsible for lymphangiogenesis (Yang et al., 2015); thus, it is a major focus in the search of anti-lymphedema drugs.

It was observed that tumors co-implanted with macrophages derived from paclitaxel-treated tumor-bearing mice regressed the phenotype of lymphatic vessels. This regression was detected to a lesser extent in paclitaxel + VEGFR-3 specific antibody (31C1)-treated counterpart mice. Although the difference was not statistically significant, this indicated that the phenotypic changes of lymphatic vessels observed in tumors after co-implantation were mediated by VEGFR-3-expressing macrophages (Alishekevitz et al., 2016).

#### 4.1.2 Radiotherapy

**FIGURE 1:**
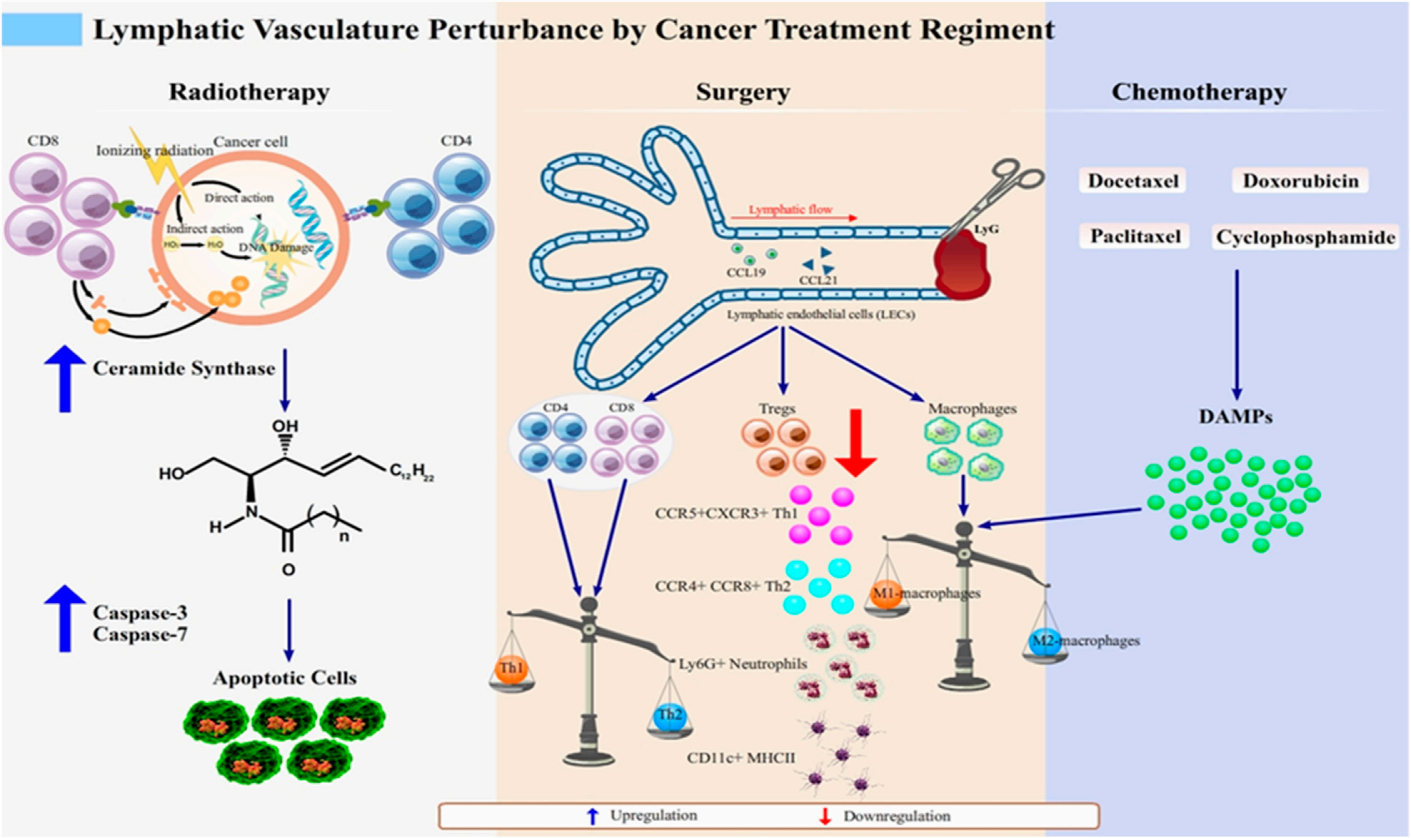
Lymphatic vasculature perturbation by anticancer regimens. Lymphedema develops after cancer treatment through various molecular events, which together highlight the disturbance of normal lymphatic physiology. Radiotherapy as an anticancer regimen enhances the expression of MHC class I molecules to improve the ability of recognition of cancerous cells by CD8^+^ T cell subset (Choi et al., 2007). However, as the proximal lymphatic vasculature, which is lined up by LECs, is unavoidably injured, the breaks in double-stranded DNA in irradiated LECs causes further damage to the mitochondria. This damage subsequently signals the *de novo* synthesis of ceramide that interacts with caspase-3 and caspase-7 for apoptosis of LECs. This initiates lymphatic dysfunction, which manifests as lymphedema (Overgaard, 2007; Warren et al., 2015; Mortezaee and Najafi, 2020).Surgery and chemotherapy share their mechanisms to induce lymphedema as the imbalanced state of pro- and anti-inflammatory macrophages. Chemotherapeutic agents such as docetaxel, doxorubicin, cyclophosphamide, and paclitaxel (Wang et al., 2017; Roh et al., 2020) induce the release of DAMPs in response to the inflammatory state of the affected tissues. DAMPs skew the Th1/Th2 balanced immune response into the Th2 response, which causes phenotypic switch of macrophages to M2 macrophages (Swaroop et al., 2015). In surgery-induced lymphedema, phenotype shifting of not only macrophage subset but also of T cells (CD4^+^, CD8^+^, and Tregs), occurs, thus mediating inflammation. T cells respond to this by secreting anti-inflammatory cytokines, IL-4 and IL-13 (Hayes et al., 2008; Degnim et al., 2012). Tregs, which phenotypically are FOXP-3 expressing CD4^+^ T cells, are downregulated, causing recruitment of various subsets such as CCR5+CXCR3+ Th1 and CCR4+CCR8+ Th2, Ly-6G + neutrophils, and CD11c+ MHCII + dendritic cells (DCs) to the inflamed tissue (Swaroop et al., 2015)^.^ Altogether, these mechanisms are attributable to lymphedema development and are promising targets for the treatment of lymphedema.

Radiotherapy produces synergetic effect with chemotherapy, and it is also a predictor of lymphedema emergence (Warren et al., 2015). The therapy is designed to destroy targeted cancer cells *via* direct or indirect pathways, as illustrated in Figure 1.

Classic dogma in radiobiology suggests that cancer cells are destroyed by ionizing radiation that can either directly damage the DNA or indirectly generate reactive oxygen species (ROS) after water radiolysis (Overgaard, 2007; Mortezaee and Najafi, 2020). Radiation breaks double-stranded DNA into two-ended DNA breaks, rendering them prone to oxidation-induced damage (Toulany, 2019; Huang and Zhou, 2020). The damage effect of radiation is time-dependent. This was modelled more than two decades ago by Mortimer et al. (1991) who irradiated Large White pigs’ skins with a single dose of 18Gy for several weekly time settings. Ischaemic and oedema massively surface in week 6–12. This corresponds to vasodilation of the dermal blood vessels. The longer the waiting, the worse impacts were seen in the skins. In week 52, the dermal thinning and subcutaneous atrophy started clinically evident (Mortimer et al., 1991).

In the molecular level, radiotherapy-associated damage induces mitochondrial ceramide synthase to mediate the *de novo* synthesis of ceramide that acts as a second messenger in apoptosis. The ceramide pathway then interact with caspase-3 and caspase-7 in intrinsic and extrinsic pathways in commencing programmed cell death (Sia et al., 2020). Caspase-3 and caspase-7 belong to the subgroup of executioner caspases that cleave the cellular target (Ponder and Boise, 2019). It was shown to be upregulated in irradiated LECs, suggesting that irradiation-induced tissue injury may sensitize LECs to apoptosis in a dose-dependent manner, leading to lymphatic dysfunction, which further surface as lymphedema (Avraham et al., 2010). Therefore, it is crucial to find approaches to formulate a low-risk lymphedema regimen for cancer patients receiving radiotherapy. However, it is obvious for majority of clinicians to primarily think about cancer treatment and any rooting potential they may bring; but these efforts are less meaningful as they create a disabling health condition.

A study by Daley et al. (2010) has paved the way for the above query. The findings of their proof-of-principle study showed beneficial effects of amifostine, a thiol derivative, in improving tissue lymphostasis and ameliorating limb lymphedema, along with no activity that favors tumorigenesis in a rodent lymphedema model. Unfortunately, reports on the use of amifostine and other substances that share similar activities in lymphedema are limited. Most reports presented amifostine as a scavenger of free radicals produced during radiotherapy in some cancer cases in humans (Rosenzweig et al., 2010; Belderbos et al., 2018), without any direct relation to the emergence of lymphedema after radiotherapy. Daley et al. successfully demonstrated the anti-lymphedema properties of amifostine, and its translation into clinical trials was expedited. Moreover, the study findings trigger redefinition and reinvention of many substances as anti-lymphedema drugs that were applied initially as a protective agent to radiotherapy.

#### 4.1.3 Surgery

Surgery is one of the well-identified inducers of lymphedema. It has been associated the most with breast cancer. There have reports exhibiting a strong association between breast cancer-associated surgery and incidence of lymphedema. As many as 33% of Australian women suffered from lymphedema in 6–18 months after their surgeries, of those 40% had long-term lymphedema (Hayes et al., 2008). Similarly, but seen in Brazilian women with breast cancers, cumulative incidence of lymphedema was observed at 13.5% within 2 years of follow up time and this increased up to 41.1% after a decade (Mortimer et al., 1991).

Several studies have demonstrated that patients who underwent surgery with axillary node removal showed a significantly higher probability of developing breast lymphedema than those who underwent only breast surgery or sentinel lymph node biopsy (Degnim et al., 2012; Boughey et al., 2014). Breast lymphedema occurs in approximately one-half of women who undergo breast or axillary surgery, that is, initially intended to remove neighboring lymph nodes and vessels (Ridner, 2013) to prevent cancerous cells from metastasizing to distant sites (Nagata et al., 2004; Zheng et al., 2018). This is expected to reduce the carrying capacity of the lymphatic system. Surgical procedures unavoidably impair the normal anatomy, which obstruct the physiological function ofthe lymphatic vessels. Moreover, surgery-induced injuries negatively impact the neighboring muscles, reducing their potential and driving force to distribute the lymphatic fluid throughout the body. In contrast, surgery may increase the blood flow rate. Stenosis in the arterial and venous axillary and brachial vessels on the limb ipsilateral to the surgery has been suggested to be responsible for the increase in flow. This change affects the contralateral limb prior to forming conditions that favor lymphedema generation (Ridner, 2013). To date, axillary lymph node dissection (ALND) and sentinel lymph node biopsy (SLNB) are standard surgical procedures for breast cancer (Gherghe et al., 2015).

Lymphatic injury following ALND might mediate local inflammation causing T cells, mainly CD4^+^ cells, to infiltrate (Nores et al., 2018). These CD4^+^ T cells produced either IL-4 or IL-13, the two most common anti-inflammatory cytokines, and were key to the severity of lymphedema (Avraham et al., 2013; Shimizu et al., 2013). In addition to CD4^+^ T cell infiltration, increase in regulatory T cells (Tregs), phenotypically characterized as FOXP3-expressing CD4^+^ T cells, was observed in lymphedema (Nores et al., 2018). Elimination of this particular subset exacerbates edema and recruits diverse subsets of CCR5+CXCR3+ Th1, CCR4+CCR8+ Th2, Ly-6G + neutrophils, and CD11c+ MHCII + dendritic cells (DCs), suggesting that Tregs also play a pivotal role in the regulation of the overall leukocyte infiltration, which marks the improvement of lymphedema (Swaroop et al., 2015).

To put the risk of surgery leading to lymphedema as low as possible, SLNB is a better alternative. Compared to breast cancer patients who underwent SLNB, those treated with ALND showed a significantly high probability of experiencing lymphedema symptoms (Miller et al., 2012). SLNB is better suited to patients with locally contralateral advanced breast cancer but not to those undergoing prophylactic mastectomy (Zhou et al., 2011).

Considering all these published reports, we concluded that the existing surgery platform, either ALND or SLNB, could not be avoided without thorough consideration. Although these reports reveal post-surgery risk of lymphedema, it is required to find a substitute therapy with similar efficiency. Previous explanation of the recruitment of Tregs post ALND to the site of injury suggest to consider Tregs as a novel curative approach for lymphedema (Gousopoulos et al., 2016). However, additional studies involving different cohorts of different primary diseases that require surgery at any stage are required. Until then, surgery remains one of the mainstay regimens for cancer, particularly breast cancer. Hence, novel strategy development against surgery-mediated lymphedema by the researchers across the globe is widely focused on providing prior protection against injury during surgery to the compartments of the lymphatic system.

### 4.2 Infections

Lymphatic filariasis is the most common pathophysiological condition caused by bacterial infection and characterized by impaired lymphatic transport affecting local immune response. Globally, more than 140 million people have been reported to suffer from lymphatic filariasis, mainly caused by *Wuchereria bancrofti* and less frequently by *Brugia malayi* (Kambhampati et al., 2016).

Interestingly, a recent case report by Hong et al. (2019) established a causative association between bacterial infection and lymphedema in the lower extremities of a patient with intestinal tuberculosis in Korea. Although an extremely rare case, it can broaden our horizon that lymphedema is not solely attributed to the adverse effects of anticancer regimens. Anti-tuberculosis drugs impaired lymphatic drainage, which resulted in ineffectiveness of the pertinent drug after the lymphatic system was damaged. These results were in line with those from previous studies by Hoda and Rab. (1974) in Korea. Both observed that although anti-tuberculosis drugs initially regressed lymph nodes and pulmonary lesions, lymphedema recurred after 7–9 months and persisted.

### 4.3 Obesity

Obesity has been considered a strong risk factor for lymphedema (Savetsky et al., 2014; Nitti et al., 2016), causing a serious clinical issue (Shimizu et al., 2013). Obesity and lymphedema have been suggested to have a reciprocal interplay (Mehrara and Greene, 2014). This was clearly observed in adipose tissues isolated from lymphedema, which were identical to fat depots in obese patients; both results from the proliferation and hypertrophy of adipocytes, which subsequently leads to the infiltration of macrophages and lymphocytes, characteristic of chronic inflammation (Zampell et al., 2012; Mehrara and Greene, 2014). At the molecular level, C/EPB-α is an essential transcription factor for determining the fate of adipocyte lineage, and adiponectin, a peptide hormone that promotes differentiation of preadipocytes into adipocytes, is upregulated in the area proximal to the region of lymphatic obstruction. The upregulation of these two proteins decreases in the distal area, according to the gradient of lymphatic stasis (Aschen et al., 2012).

In the obese state, the plasma concentration of adiponectin is decreased. The functional role of adiponectin in the lymphatic system was well described in a mouse model with ablation of tail surface lymphatic network. In this model, adiponectin-KO mice showed a greater diameter of wounded tails than that of wild-type mice. Adiponectin deficiency was associated with a low number of LECs. This phenotype can be reversed by adiponectin administration, which restores the lymphatic vasculature. *In vitro*, adiponectin promotes the differentiation and viability of LECs through the AMP-activated protein kinase (AMPK)-Akt-endothelial nitric oxide synthase pathways (Shimizu et al., 2013).

In contrast to adiponectin, leptin has been shown to be upregulated in the obese state (Korda et al., 2008; Grossmann et al., 2010). It acts through the leptin receptor in the hypothalamus to suppress food intake, but obese patients are leptin-resistant (Grossmann et al., 2010). In addition to the brain, leptin exerts its biological actions on multiple peripheral tissues, including vascular and lymphatic endothelial cells (Grossmann et al., 2010).

In the context of angiogenesis, leptin mediates neovascularization by inducing the proliferation of vascular endothelial cells and remodeling the extracellular matrix through regulation of metalloproteinases (MMPs) and tissue inhibitors of metalloproteinases (Park et al., 2001).

A high concentration of leptin, as observed in obese patients, compromises lymphatic endothelial cell homeostasis, whereas a physiological concentration of leptin maintains cellular homeostasis. A high concentration of leptin induces the expression of the proinflammatory cytokine IL-6 in human dermal lymphatic endothelial cells. Furthermore, IL-6 rescues the compromised cell proliferation and tube formation caused by high-dose leptin treatment either in an autocrine or paracrine manner. This finding suggests that leptin and IL-6 are promising intervening agents to reduce the incidence of postoperative lymphedema (Sato et al., 2016).

### 4.4 Lipedema

Lipedema is linked to lymphedema, and both are often misdiagnosed (Peled and Kappos, 2016; Buso et al., 2019). This is a clinical manifestation of bilaterally symmetrical perturbed fat deposition in the lower or upper extremities with frequent ecchymosis, and can be triggered by a minor injury (Shavit et al., 2018); lipedema is a specific disease associated to fat distribution, a distinctive feature from lymphedema.

Lipedema affects approximately 18.8% of patients with enlargement of the lower limbs and is exclusively seen in women (Forner-Cordero et al., 2012). It has been reported that 6.5% of children diagnosed with lymphedema were actually suffered from lipedema (Szél et al., 2014). Moreover, approximately 60% of lipedema cases have been reported to be genetically acquired. Although lipedema-inducing genes are yet to be inherently studied (Grossmann et al., 2010), but studies by Makinen et al. and Harvey et al. have suggested the probable genes involved in it. Mäkinen et al. (2001) showed that VEGFR-3 missense mutation played a key role in the process of subcutaneous adipose tissue thickening, whereas. Harvey et al. (2005) demonstrated that the gene PROX1 was inactivated in an adult-onset obesity and lymphatic vascular disease mouse model. Notably, both genes have been studied for their relevance in lymphedema development, strongly suggesting molecular crosstalk between lymphedema and lipedema.

Lipedema has recently received increased attention, and efforts have been made to gain a comprehensive understanding of the pathological mechanism of the disease for the development of specific intervention strategies for lipedema distinguishable from those of lymphedema. We, hitherto, are left with a major query that whether lymphedema leads to lipedema or vice versa. Future studies should be directed to unveiling the molecular crosstalk between the two.

## 5 Animal models developed to discern the acquired lymphedema

Despite the agreement that the eventual translational field of all the knowledge and concepts regarding acquired lymphedema is in the human body system, it is undeniably impossible to intentionally induce lymphedema in the human body with an expectation to derive genuine decipherment with regard to the molecular and cellular interplay that builds it. Investigators can only analyze and evaluate the pre-established lymphedema in the human body. Thus, induction and development of lymphedema is a poorly studied attribute. To comprehensively understand the development and suppression of lymphedema in the human body, primarily by the immune system, lymphedema animal models, mainly rodent models, have been proposed and used since the past three decades.

Rodents-based lymphedema model was first established by Lee-Donaldson et al. (1999) who successfully developed hind limb lymphedema in Wistar-Fuzzy rats by either microsurgical ablation (S) of the groin nodes or 4500-rad groin irradiation (R) alone as well as the combinations of the two with S followed by R and vice versa.

Tail-and hindlimb-based models are the two most common rodent models (Park et al., 2013). The tail-based model is favorable as its offers simplicity (Park et al., 2013), however the nonanatomic location and considerably small size of lymphatic vessels, to some extent, complicate surgical interventions. The hindlimb-based model, on the other hand, requires a morbid state, but the swelling resulting from the procedure is reliable, although it will not be sustained for long duration (Ghanta et al., 2016).

Our laboratory has developed a hindlimb -based murine lymphedema model by removing superficial, popliteal, and deep inguinal lymph nodes through femoral lymphatic blockade; we termed this model as SPDF-derived lymphedema. Unlike other published lymphedema models that were generated using relatively simpler methods, we focused on a sustained acquired lymphedema model; however, with regard to procedural interventions, our study required more rigorous skills to ascertain that the lymph nodes of interest were thoroughly removed and the right lymphatic vessel was blocked. We have published the details of the SPDF procedures elsewhere (Roh et al., 2017a; Roh et al., 2017b; Cho et al., 2017).

In the same year, Tran et al. (2018) established a porcine model for surgical prevention of lymphedema by inserting a central venous catheter into the jugular veins of female pigs. It was the first successful model generated through the lymphatic microsurgical preventive healing approach (LYMPHA) for lymphadenectomy. Unlike our murine model, where the mice were kept alive, this was a non-survival porcine model with some complications, including when a correlation of real-time lymphatic clearance rate with clinical lymphedema was urged to be addressed. However, Tran’s team overcame this problem by developing optically enriched fluorophores that allowed observation of lymphadenectomy using small volume injections and spectrophotometry (Tran et al., 2018). We summarize the establishment of rodent model within the last 5 years to allow an integrative understanding of purposive intricacies in model development (Table 2).

**TABLE 2:** Development of animal models of lymphedema.

Animal harnessed	Established by	Procedure	Affected body sites	Outcome
Rabbit	Tran et al. (2018)	Skin denudation with destruction of the lymph channels by microsurgery	Right ear	Significant ear thickness on day 7–15 post intervention
Rat	Daneshgaran et al. (2019)	Surgical dissection of the superficial cervical lymph nodes and deep cervical lymph nodes followed by irradiation	Head and neck	Head and neck swelling and subcutis thickness were significantly elevated along with increased expression of TGF-β1
Rat	Yang et al. (2014)	Inguinal and or popliteal lymph node removal and the combination along with irradiation	Lower limb	Lymphedema was reliably developed in the lower limb with minimum morbidity within 4 months
Mouse	Weiler et al. (2019)	Circumferential incision spanning dermal layer of the tails of the pre-obesity induced mice	Tail	Obesity exacerbated acquired lymphatic pump failure after lymphatic vessels were injured
Rat	Will et al. (2020)	Subcutaneous dissection and deep lymphatics and lymph nodes skeletonization	Hindlimb	Acquired lymphedema was successfully developed with no complications. The SL was sustained up to 48 days after surgery
Mouse	Ogino et al. (2020); Hayashida et al. (2017)	X-irradiation and circumferential incision in the inguinal region to the muscle layer	Hindlimb	LEC promotion-mediated lymphedema was reduced following the adipose-derived stem cell (ADSC) transplantation. In addition, the number of lymphatic vessels was increased through intussusceptive lymphangiogenesis
Mouse	(Roh et al., 2017a; Roh et al., 2017b)	Removal of superficial, popliteal, and deep inguinal lymph nodes and blockade of the femoral lymphatic system, termed as SPDF surgery	Hindlimb	Lymphedematous enlargement lasted for 21 days post-SPDF surgery

As listed in Table 2 and many other published reports that have not been included in the table, it is clear that rats and mice are the two most exploited rodents for lymphedema models. Despite the technical drawback of small-sized lymphatic compartments, the reproducibility of lymphedema in rats and mice that mimic the actual clinical symptoms observed in patients might have attracted the researchers to use these models. The models were established to justify specific interests of the researchers; hence, the interventions were quite different. However, we can highlight that, despite the differences in surgical intervention strategy, lymphatic drainage should be firmly perturbed to allow manifestation of clinical outcomes of interest. These differently built models also provide diverse directions for future studies, which are expected to intersect at a point where an alleviative strategy needs to be successfully formulated to suppress the emergence or relapse of lymphedema as either chronic individual disease or comorbidity and to improve the quality of life of the affected patients.

## 6 Phytochemicals-based substances against lymphedema

Despite the prevailing information about lymphedema induction and development, its intervention strategy is yet to be formulated. Currently, there is no licensed medicine for lymphedema. This is so unfortunate considering that the number of patients suffering from lymphedema, particularly the patients with breast cancer, is growing by multiple times (Pappalardo et al., 2021). Anti-inflammatory capacity, thus far, is seen as an underlying mechanism rendering some prior chemical-based drugs are recommended to treat lymphedema (Forte et al., 2019).

In 2004, benzopyrone, which is generally prescribed to reduce vascular permeability, was observed to be promising as an anti-lymphedema drug because of its ability to enhance macrophage activity and accelerate the lysis of proteins to block fibrosis in the lymphodematous limbs. Unfortunately, this claim was not strongly supported by the results of a meta-analysis; hence, its effectiveness remains questionable (Badger et al., 2004). A decade later, ubenimex, a synthetic organic compound, which has the ability to promote lymphatic flow similar to that observed with benzopyrone, entered a phase 2 clinical trial. However, ubenimex is only available in Japan and the United States (Chiu et al., 2020). Deupirfenidone (LYT-100), a synthetic chemical compound, is the most recently developed anti-lymphedema drug in phase 2a clinical trial, and studies on its safety, pharmacokinetics, and tolerability are being conducted. Deupirfenidone is principally a deuterated form of pirfenidone, which is known to possess inhibitory activity on the release of collagen and cytokines (https://adisinsight.springer.com/drugs/800038358). Pirfenidone alone has been used to treat idiopathic pulmonary fibrosis (Sgalla et al., 2018; Cottin et al., 2019), which led to a more recent hypothesis that pirfenidone might also work to alleviate pulmonary-related deterioration in COVID-19 patients (George et al., 2020; Seifirad, 2020).

Moreover, Chen et al. (2015) demonstrated that calycosin and gallic acid, chemical derivatives from *Radix astragal* and *Radix Paeoniae*, respectively, synergistically induced the expression of leukotriene B4 dehydrogenase (LTB4DH), which attenuated leukotriene B4 (LTB4) (Cheng et al., 2015). LTB4 belongs to a family of eicosanoid inflammatory mediators involved in the innate immune response and is highly associated with the pathophysiology of lymphedema (Jiang et al., 2018). In a surgery model of lymphedema in mouse, LTB4 was observed to be elevated, which was counterproductive towards the repair of the lymphatic tissues affected by surgery; hence, blocking of LTB4 has been observed to negate this effect (Tian et al., 2017). In addition to calycosin and gallic acid, Goreisan has been implicated in alleviation of lower-limb lymphedema (LLL) in patients with gynecologic cancers who underwent pelvic lymphadenectomy. Goreisan is a traditional Japanese herb comprising “bukuryo” (hoelen), “takusha” (*Alismatis rhizoma*), “sojutsu” (*Atractylodes lanceae rhizoma*), “chorei” (polyporus), and “keihi” (cinnamon bark). This herb is available in China and Korea. LLL patients who received Goreisan showed a remarkable reduction in extracellular water (ECW)/total body water (TBW) ratio, suggesting that the herb ameliorated the excessive accumulation of extracellular fluid that restricted free physical movement of extremities proximal to the lymphadenectomy-affected sites (Yoshikawa et al., 2020).

Our laboratory contributed to this global effort by demonstrating that sulfuretin extracted from *Rhus verniciflua* is a promising anti-lymphedema agent (Roh et al., 2017a). *Rhus verniciflua* belongs to the family *Anacardiaceae* and is commonly known as the lacquer tree, which is indigenous to Korea, Japan, and China. This has been used as an alternative herbal medicine to cure patients with diabetes and stomach-related diseases (Choi et al., 2007). Oral administration of the extract has shown to reduce adipocyte deposition in lymphedematous tissue in SPDF-induced lymphedema-bearing murine model. In contrast, the expression of VEGFR-3, a marker of lymphangiogenesis, was induced after sulfuretin treatment (Roh et al., 2017a). Similarly, saponins extracted from *Panax notoginseng* (PNS), which are commonly used in traditional Chinese medicine for cardiovascular and cerebrovascular diseases, promote lymphangiogenesis in a VEGF-C/VEGFR-3 fashion in zebrafish. Furthermore, ERK1/2, PI3K, and P38 MAPK signaling pathways were deemed to be pivotally involved. This widened the horizon on suitable therapeutic agents for acquired lymphedema (Sgalla et al., 2018) which previously and even to date is thought to be a frustrating dead-end. Moreover, all these studies have stimulated additional studies on plant-derived chemicals exerting anti-lymphedema activity. The above descriptions are listed in Table 3.

**TABLE 3:** Some herbals/phytochemical substances with promising anti-lymphedema properties.

Substance	Plant of origin	Anti-lymphedema proposed mechanism	Reported by
Calycosin	*Radix astragal*	Attenuation of leukotriene B4 (LTB4: member of eicosanoid inflammatory mediator family) by inducing the production of LTB4 dehydrogenase (LTB4DH)	Cheng et al. (2015)
Gallic acid	*Radix Paeoniae*		
Goreisan	Mixture of Hoelen, *Alismatis rhizoma*, *Atractylodes lanceae rhizoma*, polyporus, and cinnamon bark	Amelioration of excessive deposition of extracellular matrix	Yoshikawa et al. (2020)
Sulfuretin	*Rhus verniciflua*	Reduction of adipocyte deposition by improving lymphangiogenesis	Roh et al. (2017a)
Saponins	*Panax notoginseng* (PNS)	Lymphangiogenesis promotion	Li et al. (2016)

## 7 Preventive management of acquired lymphedema

Despite the well-identified of clinical risk factors for lymphedema, firm scientific evidence for its preventive methodology is lacking, which lead anxiety for those affected patients. Regardless, a common sense approach is deemed to be adoptable while optimal prevention platform are yet to find its best structure (Cemal et al., 2011). The prevention strategy covers all steps from measuring the risk factors to preventing the accumulation of fibro-adipose tissue produced by chronic lymph stasis (Prevention of Lymphedema, 2022) which is best exemplified in breast cancer therapy-related lymphedema. So far, among other cancer types, breast cancer is regarded as the highest contributor to lymphedema (American Cancer Society, 2022). As the first step of prevention, BMI, lymph nodes status and radiotherapy effects for those who have undergone radical mastectomy are needed to be carefully evaluated by clinicians to avoid complication (Hua-Ping et al., 2012). As reported by Jammallo et al. (2013), pre- and post-operative BMI were significantly associated with risk of lymphedema. Either a pre-operative BMI ≥30 (Jammallo et al., 2013; Can et al., 2016) or a huge fluctuation, regardless a 10 pounds-gain or loss increases risk of lymphedema markedly (Jammallo et al., 2013). Like BMI, the number of metastatic lymph node is also regarded as one of determinant lymphedema inducers. It was evident as the number of patients developing lymphedema after receiving breast conserving surgery (BCS) in combination with ALND was not statistically significant compared to that of patients with no lymphedema (Can et al., 2016). Moreover, other study demonstrated that prophylactic lymphovenous anastomosis (LVA) conducted after lymphadenectomy was potentially lower the risk of cancer-related lymphedema without negating the cancer treatment (Ciudad et al., 2022). In gynaecological cancer cases, early prevention of complex decongestive therapy (CDT) combined with physical exercise prevents lower extremity lymphedema post operation (Wu et al., 2021). CDT is designed to alleviate the swelling, enhance the condition of skin, improve mobility, reduce the risk of infection and optimize the quality of health (OncoLink Team, 2022). CDT encompasses two-phase approach:1) phase I (intensive) is aimed at mobilizing, reducing the congested protein-enriched fluid softening the connective tissue and 2) phase II (maintenance) is aimed at preservering results derived from phase I (Michopoulos et al., 2020). Up to date, CDT is referred as the gold standard of lymphedema treatment (LymphCare, 2021). Although specific mechanisms are yet to be clear and so far the study of CDT are likely to be in randomized trials, this has been widely shown to alleviate lymphedema in different patients’ cohorts (Yesil et al., 2017; Omidi et al., 2020; Wu et al., 2021). This suggests that, although finding an effective preventive startegy is yet to see its ends, CDT is feasible to implement.

## 8 Discussion

Acquired lymphedema, despite its well-characterized clinical symptoms, remains a major problem to solve. This emergence reduces the quality of life of affected patients and worsens when a relapse recurs. Several studies have shown that relapsed lymphedema is almost impossible to reverse. As an adverse effect to cancer treatment, lymphedema exhibits multi-complex molecular and cellular orchestration, which could either favor or unfavor tumorigenesis and lymphangiogenesis. This is a favorable mechanism to alleviate the excess lymphatic fluid accumulation preceded by lymphatic injury. However, lymphangiogenesis is unfavorable for tumorigenesis suppression. This is of conflicting profiles, which demands further in-depth investigation of curative agents against cancers that concomitantly act as preventive agents against potentially developed lymphedema. To discern intricacies in lymphedema, diverse types of animal models have been developed, with murine-based model being the most commonly exploited. Although models do not precisely and frequently address all our queries, they resemble the best to human molecular pathways; hence, they are able to provide evidences related to the anticancer mechanism. This is also the most feasible living tool to evaluate loss-gain functions, as consequences of up/down regulations of well-known lymphedema-associated genes, mainly *VEGF-C/VEGFR-3, TFG-β1, and C/EBP-α/β*, through which clear functions of the pertinent genes can be mapped firmly. In addition, the probability of negation functions of the associated genes as well as crosstalk predisposition can be evaluated. When this is contraindicated to the overall health status and quality of life post treatment, a strategy to prevent further detrimental body reactions can be specifically formulated. Phytochemical-based substances generally emerge as a suitable approach to this debilitating comorbid disease. Their anti-lymphedema properties, which are mediated *via* different pathways, are attractive for further exploration.
